# Integrin linked kinase and threonine tyrosine kinase modulate TCR signaling

**DOI:** 10.1038/s41598-025-99331-y

**Published:** 2025-04-24

**Authors:** Vivien Caillens, Eva Boisel, Alycia Ouksel, Mathilde Nugue, Irini Evnouchidou, Loredana Saveanu

**Affiliations:** 1 Centre de Recherche sur l’Inflammation, U1149 INSERM, Faculté de Médecine X Bichat, 16 rue Henri Huchard, Paris, 75018 France; 2 CNRS ERL8252, Paris, France; 3 Université de Paris-Cité, Site Xavier Bichat, Paris, France; 4 Inflamex Laboratory of Excellence, Paris, France

**Keywords:** Antigen T cell receptor, T cell activation, ILK, TTK, Immunology, Signal transduction

## Abstract

T cell activation is critical for adaptive immunity, helping to protect the body from infection and tumors. A key step in this activation is signal transduction downstream of the T cell antigen receptor. This signaling involves several steps, with early ones occurring at the plasma membrane and others that occur later, after TCR internalization. The late steps in TCR signaling remain poorly understood. Since the TCR can signal after its internalization, we postulated that kinases abundantly expressed in T cells may regulate TCR signaling. This study focuses on two such enzymes: integrin-linked kinase (ILKs) and threonine-tyrosine kinase (TTKs), whose involvement in TCR signaling has not been previously studied. Using specific depletion of TTK and ILK by lentiviral shRNA, we show that in the absence of ILK and TTK, the early steps of TCR signaling are strongly enhanced, while IL-2 production by activated T cells is strongly decreased. These findings are relevant because TTK and ILK are both important targets in oncology, and our results show that their inhibition affects the activation of T cells, which play an essential role in anti-tumor defense.

**Supplementary Information:**

The online version contains supplementary material available at 10.1038/s41598-025-99331-y.

## Introduction

Adaptive immune response strongly depends on T cell mediated immunity. T cells, *via* their antigen receptor (TCR), are able to recognize non-self and eliminate infected cells and cancer cells expressing neo-antigens. This recognition is based on the interaction of the TCR with antigenic peptides bound to major histocompatibility complex (MHC) molecules on antigen presenting cells (APCs).

The TCR is present at the plasma membrane as an octameric complex formed by αβ heterodimers that recognize the antigen and CD3ε, γ, δ and ζ chains that trigger intracellular signaling cascades that are well characterized for the first minutes after TCR engagement^1^. These signaling cascades start with the phosphorylation of immunoreceptor tyrosine-based activation motifs (ITAM) of the CD3 chains by the Src kinase Lck. Phosphorylated ITAMs are docking sites for the kinase ZAP70, which phosphorylates then the linker for activation of T cells (LAT) on tyrosine residues. Phosphorylated LAT recruits PLCγ1 and downstream signaling adaptors such as Grb2, Gad and SLP76, leading to Ca^2+^ increase and activation of Rho GTPases and MAPKs^2^. These early signaling events peak within the first minutes of TCR activation and are followed by endocytosis of activated TCR, which was initially considered to be a mechanism of signal termination through degradation of the activated receptor^3^.

However, the fact that TCR internalization serves exclusively to abolish TCR signaling was somewhat challenged by the discovery that endocytosis is required for antigen-induced T cell activation. In this context, it has been shown that cells deficient in dynamin-2, a large GTPase required for endocytosis, have increased TCR expression at the plasma membrane, but decreased proliferation due to reduced mTORC1 signaling downstream of TCR activation^4^. This led to the hypothesis that internalized TCR continues to signal from endocytic compartments, as previously suggested by the visualization of signaling competent components of TCR signalosomes in intracellular vesicles^3,5^. The hypothesis of TCR endosomal signaling was later strengthened by the visualization, using a fluorescence resonance energy transfer (FRET) reporter, of the interaction of the CD3ζ chain of the TCR complex with the ZAP70 kinase at the level of endosomes, described by the small GTPase Rab5^6^ or by IRAP (Insulin Responsive AminoPeptidase) and Stx6 (syntaxin 6)^7^. Using models of T cell IRAP-deficient mice, we have shown that the intracellular pool of the CD3ζ chain, and its association at this level with signaling molecules, are essential for T cell survival in the periphery, as well as for their ability to infiltrate tumors and control their growth in an antigen-specific manner^7^.

This association at the endosomal level of the CD3ζ chain with the classical molecules of TCR-mediated signaling, up to 30 min after TCR internalization, suggests that the late stages of signaling may involve other protein kinases not previously associated with TCR signaling.

Therefore, we hypothesized that other protein kinases that are highly expressed in T cells may be involved in signaling downstream of the TCR. To test this hypothesis, we focused on integrin-linked kinase (ILK) and threonine-tyrosine kinase (TTK). In the case of ILK, our choice was motivated by the fact that mice deficient in ILK in T cells show a very strong phenotype, with almost complete ablation of T cells in the periphery^8^. This suggests that ILK is somehow required for T cell survival. On the TTK side, our choice was motivated by the fact that TTK was first identified in T cells and that this kinase appears to play a role in T cell proliferation^9^, which is one of the major outcomes of TCR activation^10^.

As suggested by its name, ILK was discovered in 1996 by a two-hybrid assay in which the cytosolic domain of the β1 integrin was used as a bait^11^. Subsequent studies have shown that ILK is a ubiquitous protein involved in the regulation of multiple cellular processes, including cell proliferation and survival^12^. Analysis of ILK-deficient T cells revealed a defect in their response to the chemokines CXCL12 (stromal cell-derived factor SDF-1α) and CCL19 (macrophage inflammatory protein MIP-3β), and increased apoptosis of ILK-deficient cells upon stress. These defects have been attributed to a decrease in Akt kinase and Rac activity^8^, but to our knowledge, TCR signaling in the absence of ILK has never been carefully investigated.

In contrast to ILK, which has been studied to some extent in T cells^8^, TTK, although initially identified in T cells where it is highly expressed^9,13^, has been studied almost exclusively in the context of carcinogenesis, where its effects in facilitating cell proliferation make it a preferred target for anti-proliferative therapy in oncology^14^.

Overall, ILK and TTK seemed to us to be two potential candidates for involvement in TCR downstream signaling. We decided to inactivate them in T cells and see how the classical TCR downstream signaling cascade was affected. Our results show that these two enzymes are negative regulators of TCR signaling and at the same time are essential factors for IL-2 production downstream of TCR activation.

## Results

### ILK depletion increases proximal TCR signaling

As previously mentioned, ILK is ubiquitously expressed and regulates multiple cellular processes, including cell proliferation and survival^12^. The ILK protein structure includes an amino-terminal ankyrin repeat domain that interacts with several adaptor proteins, followed by a PH-like domain that binds phosphoinositides, such as phosphatidylinositol 3,4,5-triphosphate generated by class I PI3K. The carboxy-terminal domain of the protein contains the kinase catalytic domain. This domain is required for the phosphorylation of the serine 473 residue of AKT, resulting in AKT activation, and for the phosphorylation of the serine 9 residue of GSK3β (Glycogen synthase kinase 3 β), resulting in GSK3β inhibition^12,15^. AKT activation by ILK leads to mTORC activation, which explains why ILK-deficient T cells show increased apoptosis^8^. In addition to the survival defect, ILK-deficient cells have a significant reduction in chemotaxis towards the chemokines CXCL12 and CCL19^8^.

**Fig. 1:**
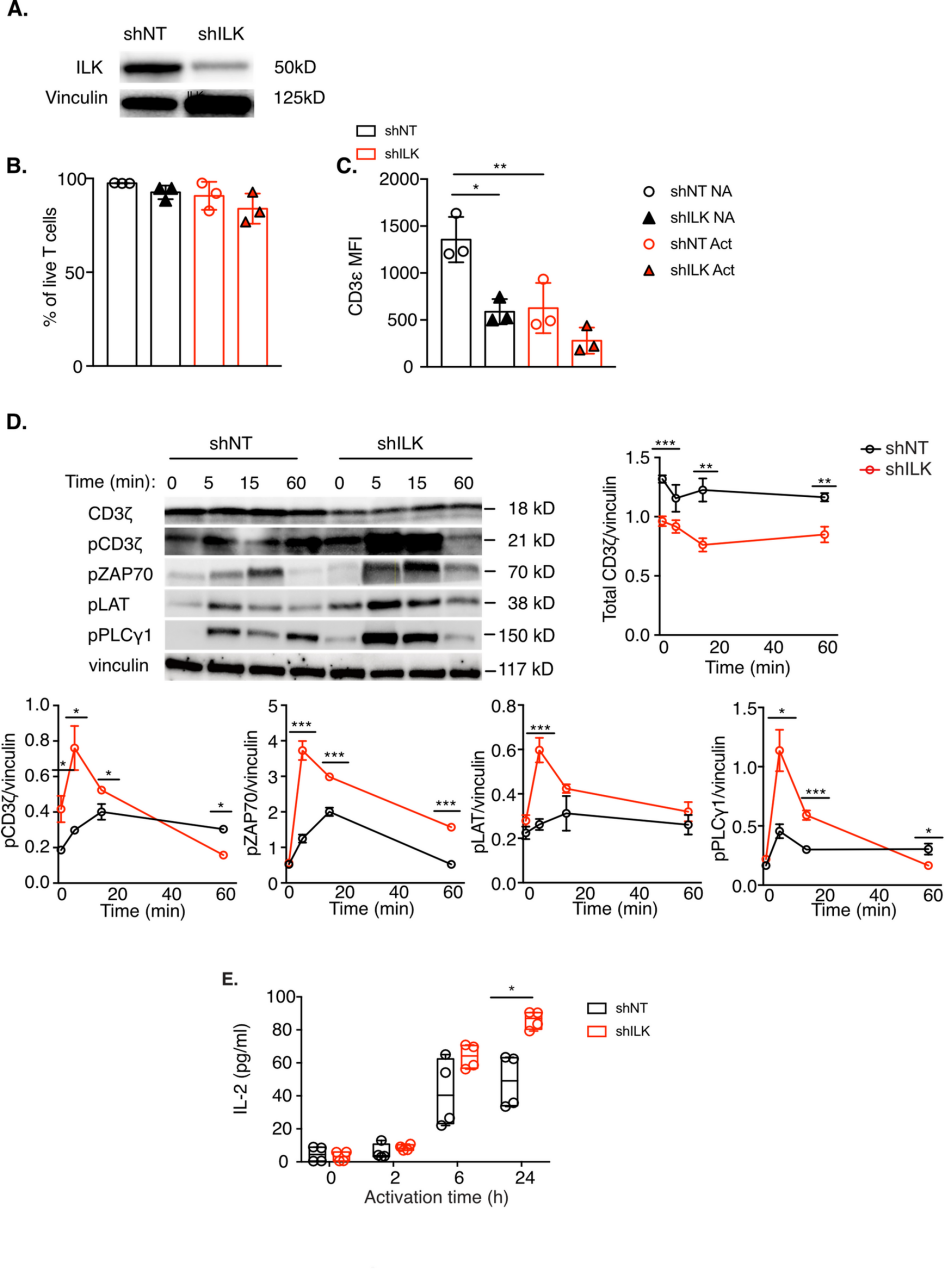
ILK depletion affects TCR signaling. **A**. Jurkat T cells were transduced with lentiviruses expressing shRNA specific for ILK (shILK) and control, non-targeting (shNT) shRNA. ILK depletion was verified by immunoblotting. **B-C.** Jurkat T cells were transduced with lentiviruses expressing control (shNT) or ILK specific (shILK) shRNA. Cell viability (**B**) or cell surface TCR levels (**C**) were analyzed by flow cytometry. **D-E**. Control (shNT) and ILK-depleted T cells were activated with anti-CD3ε/anti-CD28 antibodies for the indicated time points. TCR signaling was monitored by immunoblot against the total CD3ζ and the phosphorylated and active forms of: CD3ζ, ZAP70, LAT and PLCγ1 (**D**), while IL-2 production was measured by ELISA (**E**). The graphs in (**D**) show the quantification of the immunoblots using vinculin as a loading control. The graph in (**E**) shows 2 independent experiments in duplicates. Statistical analysis: Student t test, unpaired, * *p* < 0.05, ** *p* < 0.01, *** *p* < 0.001, **** *p* < 0.0001.

**Fig. 2:**
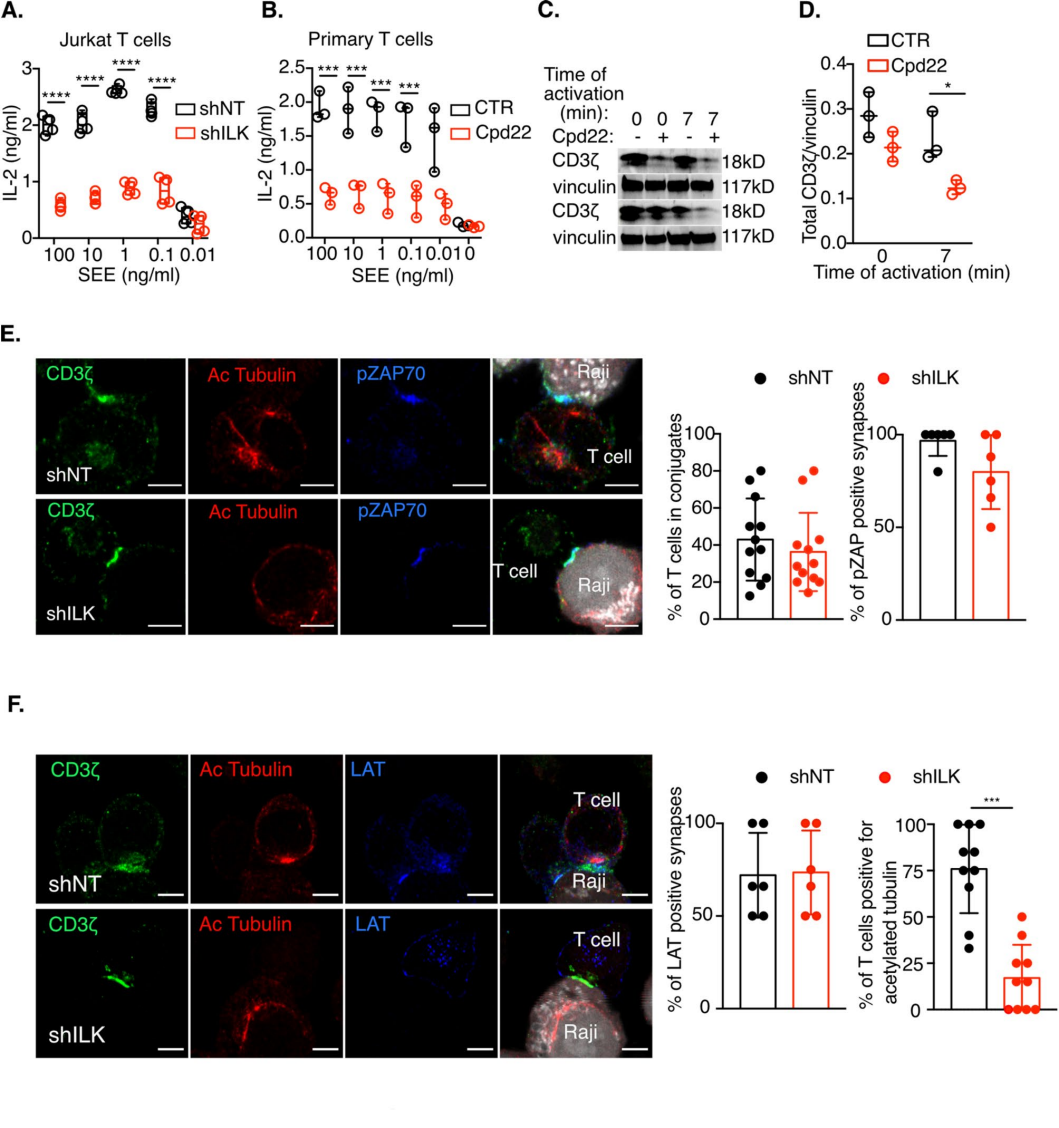
ILK deficiency affects IL-2 secretion, but not immune synapse formation upon SEE activation. **A.** Jurkat T cells were transduced with lentiviruses expressing ILK-specific (shILK) and control, non-targeting (shNT) shRNA and incubated with SEE-pulsed Raji B cells for 6 h. IL-2 secretion was measured by ELISA. The results represent a pool of 4 independent experiments. **B.** Human primary T cells isolated from fresh blood were activated with SEE-pulsed Raji B cells in the absence (CTR) or the presence of the ILK inhibitor Cpd22 and IL-2 secretion was measured by ELISA 6 h later. The results represent a pool of 3 experiments with T cells isolated from 3 different donors. Each point corresponds to one donor. **C.** Human primary T cells were incubated with the ILK inhibitor Cpd22 for 1 h and CD3ζ expression was quantified by immunoblot with and without TCR activation by anti-CD3 and anti-CD28 antibodies (7 min). **D**. The graph shows the CD3ζ quantification of the experiment shown in **C**, for T cells isolated from 3 different donors. Each point corresponds to a donor. **(E)** ILK-depleted Jurkat T cells (shILK) and control cells (shNT) were incubated with SEE-pulsed Raji B cells, fixed and stained with anti-pZAP70 and anti-Acetylated tubulin antibodies. The graphs on the right show the percentage of T cells forming conjugates with B cells and the percentage of pZAP70 positive synapses. Scale bars = 5 μm. Each dot represents the quantification of a microscope field from 2 independent experiments. **(F)** ILK-depleted Jurkat T cells (shILK) and control cells (shNT) were incubated with SEE-pulsed Raji B cells, fixed and stained with anti-LAT and anti-Acetylated tubulin antibodies. The graphs on the right show the percentage of LAT positive synapses and the percentage of T cells positive for acetylated tubulin. Scale bars = 5 μm. Each dot represents the quantification of a microscope field from 2 independent experiments. Statistical analysis: Student t test, unpaired, * *p* < 0.05, ** *p* < 0.01, *** *p* < 0.001, **** *p* < 0.0001.

Considering these previous findings, we decided to study the role of ILK in TCR signaling and to this end, we depleted ILK by lentiviral shRNA in Jurkat T cells (Fig. 1A). ILK depletion did not affect T cell survival, either in basal conditions, or after TCR activation (Fig. 1B), but it induced a significant decrease in the level of the TCR complex expression at the cell surface, both at basal level and after activation (Fig. 1C). Consistent with the reduction in cell surface TCR levels, ILK-depleted cells showed an important decrease in the total amount of CD3ζ chain by immunoblot (Fig. 1D). Unexpectedly, despite the low levels of the CD3ζ chain in the absence of ILK, upon activation by anti-CD3ε and anti-CD28 antibodies, the ILK-depleted cells showed an increase in TCR signaling, as illustrated by increased phosphorylation of CD3ζ, ZAP70, LAT and PLCγ1 (Fig. 1D), as well as increased secretion of IL-2 (Fig. 1E). Nevertheless, when the TCR was activated with Raji B cells loaded with the SEE superantigen, IL-2 secretion was strongly reduced in the absence of ILK (Fig. 2A), demonstrating that ILK negatively regulates TCR signaling while positively regulating the superantigen-induced signaling cascade, which depends on TCR but also on unknown G protein-coupled receptors (GPCRs)^16,17^. The strong reduction in IL-2 production and in the total amount of the CD3ζ chain upon SEE activation was confirmed in human primary T cells treated for 1 h with the ILK inhibitor Cpd22^18^ (Fig. 2B–D). The inhibition of ILK in human primary CD8+ T cells induced a slight but not significant reduction in CD25, CD69 and Ki-67 activation and proliferation markers (Supplementary Figs. 1 and 2) and a significant reduction in the production of IFNγ, TNFα and IL-2 (Supplementary Fig. 2). Inhibition of ILK in primary CD4 + T cells had a similar effect as in CD8 + cells, although to a lesser extent (Supplementary Fig. 3). The decrease in cytokine production upon SEE activation in ILK-depleted Jurkat T cells was not due to a defect in immune synapse formation since ILK-depleted Jurkat cells were able to form immune synapses with SEE-pulsed Raji B cells (Fig. 2E, F). The recruitment of CD3ζ, ZAP70 and LAT to the immune synapse was normal in the absence of ILK (Fig. 2E, F). In addition, as previously published in other cell types^19^, ILK deficiency in T cells appears to affect microtubule stability, as suggested by a sharp reduction in the acetylated tubulin staining in ILK-depleted cells (Fig. 2F). The reduction in acetylated tubulin may be explained by the increased TCR signaling in the absence of ILK, since microtubule stability depends on the acetylation status of tubulin, and it has been shown that TCR binding induces transient deacetylation of tubulin^20^. Taken together, these results demonstrate that ILK has a previously unappreciated role in TCR signaling in addition to its role in integrin signaling. In the absence of ILK, TCR signaling as well as IL-2 production after anti-CD3/anti-CD28 activation are upregulated, but surprisingly, IL-2 production upon SEE activation is strongly decreased.

### TTK depletion affects TCR signaling and IL-2 secretion

**Fig. 3:**
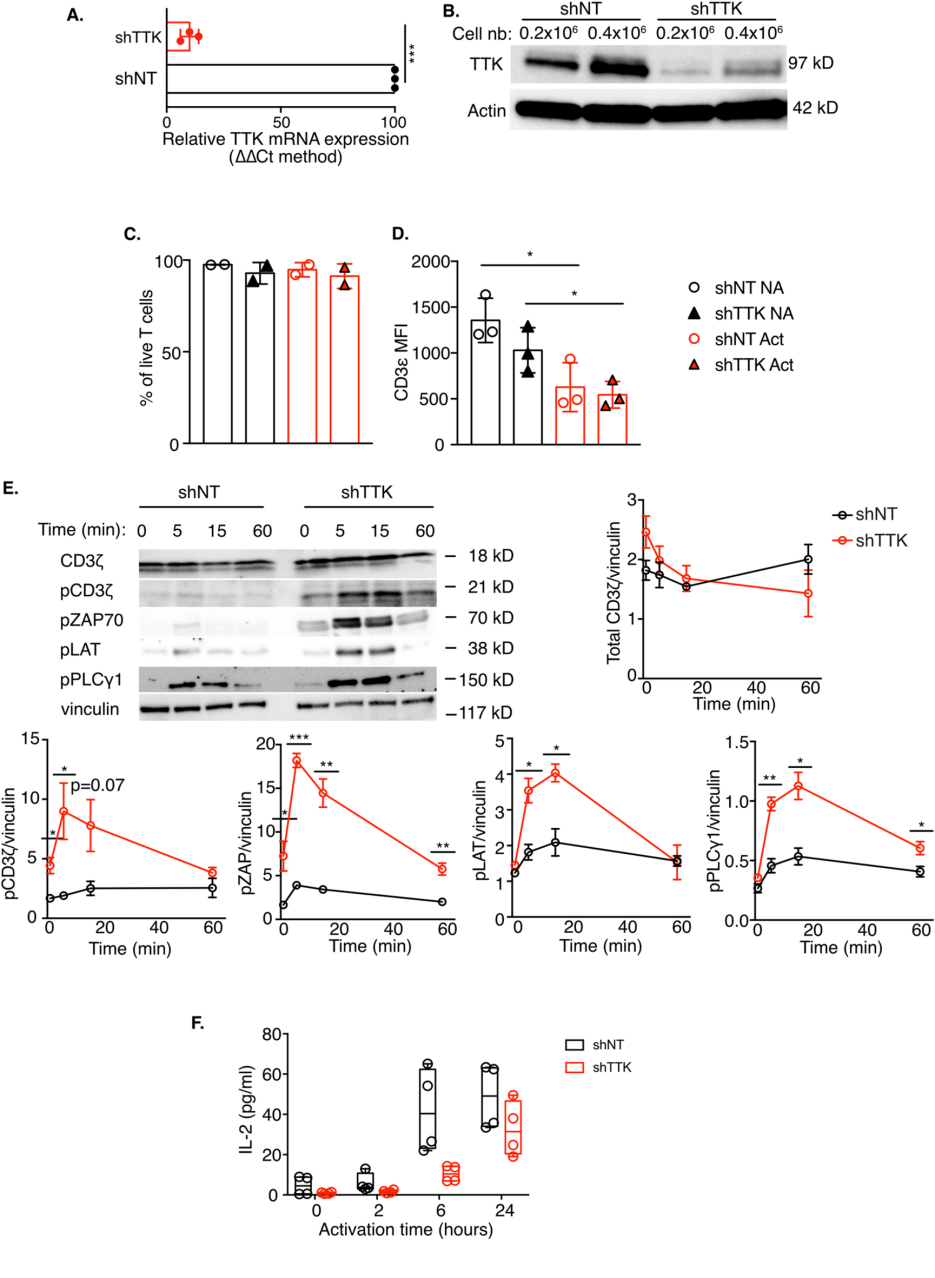
TTK depletion affects TCR signaling. **A, B** Jurkat T cells were transduced with lentiviruses expressing anti-TTK (shTTK) and control, non-targeting (shNT) shRNA and TTK depletion was verified by qRT-PCR using GAPDH as reference (**A**) or immunoblot using Actin as reference (**B**). **C, D** Jurkat T cells were transduced with lentiviruses expressing control (shNT) or anti-TTK (shTTK) shRNA. Cell viability (**C**) or cell surface TCR level (**D**) were analyzed by flow cytometry at steady state (NA) or after activation with SEE-loaded Raji B cells. **E, F**. Control (shNT) and TTK-depleted T cells were activated with anti-CD3ε/anti-CD28 antibodies for the indicated time points. TCR signaling was monitored by immunoblot against the total CD3ζ and the phosphorylated and active forms of: CD3ζ, ZAP70, LAT and PLCγ1 (**E**), while IL-2 production was measured by ELISA (**F**). The graphs in (**E**) show the quantification of the immunoblots using vinculin as a loading control. The graph in (**F**) shows 2 independent experiments in duplicates. Statistical analysis: Student t test, unpaired, * *p* < 0.05, ** *p* < 0.01, *** *p* < 0.001, **** *p* < 0.0001.

**Fig. 4:**
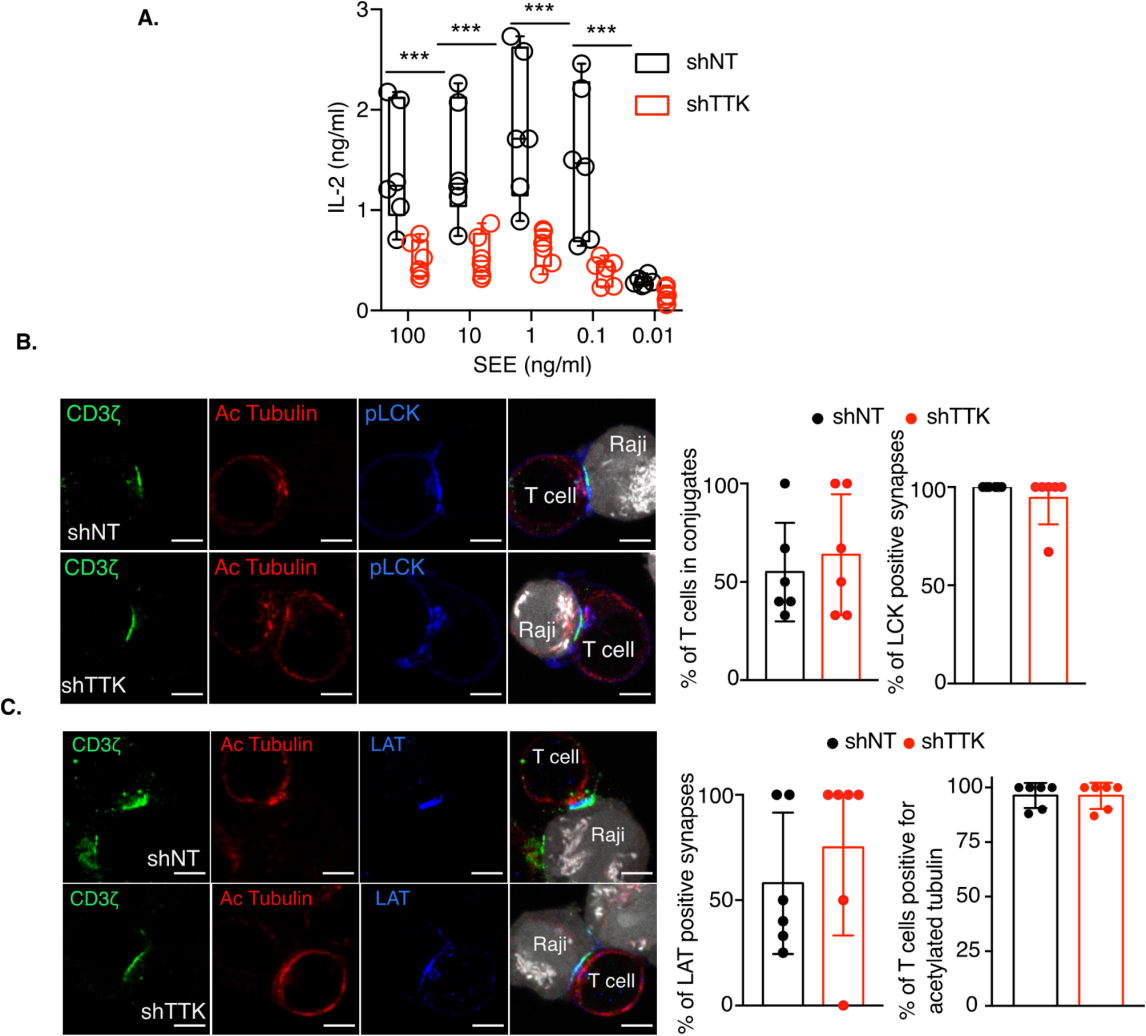
TTK depletion affects IL-2 production, but not the formation of immune synapses upon SEE activation. **(A)** Control (shNT) and TTK-depleted T cells were incubated with SEE-pulsed Raji B cells for 6 h and IL-2 secretion was measured by ELISA. The results represent a pool of 3 independent experiments. **(B)** TTK-depleted Jurkat T cells (shTTK) and control cells (shNT) were incubated with SEE-pulsed Raji B cells, fixed and stained with anti-pLCK and anti-Acetylated tubulin antibodies. The graphs on the right show the percentage of T cells forming conjugates with B cells and the percentage of pLCK positive conjugates. **(C)** TTK-depleted Jurkat T cells (shTTK) and control cells (shNT) were incubated with SEE-pulsed Raji B cells, fixed and stained with anti-LAT and anti-Acetylated tubulin antibodies. The graphs on the right show the percentage of LAT positive synapses and the percentage of T cells positive for acetylated tubulin. Scale bars = 5 μm. Each dot represents the quantification of a microscope field from 2 independent experiments. Statistical analysis: Student t test, unpaired, * *p* < 0.05, ** *p* < 0.01, *** *p* < 0.001, **** *p* < 0.0001.

TTK, also known as Mps1, can phosphorylate serine, threonine and tyrosine residues and it is abundantly expressed in highly proliferative cells^9^. TTK was first identified in T cells, by a screening with anti-phosphotyrosine antibodies^9^, and its expression has been shown to be induced by IL-2^13^. However, the role of this kinase in T cells has not been further investigated. To investigate the role of TTK in TCR signaling, we depleted TTK in Jurkat T cells using TTK-specific shRNA (Fig. 3A, B). Depletion of TTK did not affect T cell viability (Fig. 3C), TCR complex levels at the plasma membrane or the internalization of activated TCR (Fig. 3D). As expected from the normal TCR levels at the plasma membrane, the total amount of CD3ζ was not affected by TTK depletion (Fig. 3E). Similar to ILK depletion, TTK depletion induced a strong increase in TCR downstream signaling, as shown by the increased phosphorylation levels of CD3ζ, ZAP70, LAT and PLCγ1 (Fig. 3E). Surprisingly, and contrary to what was shown with ILK, despite this increased phosphorylation of TCR signaling partners, TTK-depleted T cells show slightly reduced levels of IL-2 compared to control cells transduced with a lentivirus encoding a non-targeting shRNA after anti-CD3/anti-CD28 activation (Fig. 3F). However, similar to ILK depletion, TTK depletion also leads to reduced IL-2 secretion after SEE activation (Fig. 4A). The number of T cells- B cell conjugates was similar between control and TTK depleted cells (Fig. 4B, C).

In conclusion, TTK depletion resulted in a phenotype on TCR signaling quite similar to ILK depletion, i.e. increased phosphorylation of key members of the early TCR signaling cascade upon anti-CD3/anti-CD28 antibody activation, but a defect in IL-2 secretion upon both anti-CD3/anti-CD28 or superantigen activation of T cells.

## Discussion

Based on the discovery that the TCR continues to signal from endocytic compartments after its endocytosis^4,6,7^, we decided to investigate the role of protein kinases highly abundant in T cells that have not been previously studied in the context of TCR signaling, such as ILK and TTK, two serine threonine kinases. Our decision to study TCR signaling in the absence of ILK was motivated by the previously published phenotype of T cells from ILK-deficient mice^8^. ILK-deficient T cells were reported to have reduced survival due to increased cell death of double positive thymocytes and reduced chemotaxis to the chemokines CXCL12 and CCL19, but TCR signaling was not investigated in these cells. Our study shows that ILK depletion or inhibition does not affect T cell viability (Fig. 1A), but affects the total amount of TCR levels at the plasma membrane (Fig. 1C) and the total amount of TCR CD3ζ chain (Fig. 1D). The finding that ILK controls the cellular levels of the CD3ζ chain reveals a new function for this protein in T cells in addition to its previously reported role in the regulation of chemotaxis^8^. The molecular mechanisms by which ILK controls the cellular level of the CD3ζ chain are not known, but one possibility that merits future investigation is the potential role of ILK in microtubule stabilization, as reported in other cell types^19,21^. In support of this hypothesis, ILK-depleted cells showed reduced levels of acetylated tubulin, which is considered as a marker of stabilized microtubules (Fig. 2E, F). By regulating microtubule stability in T cells, ILK may contribute to CD3ζ chain trafficking, which is dependent on the microtubule network, as demonstrated by the phenotypes of End-binding protein 1 (EB1)^22^ and dynein-depleted T cells^23^.

Unexpectedly, despite low levels of the TCR complex at the plasma membrane in ILK-depleted cells, the phosphorylation of CD3ζ, ZAP70, LAT and PLCγ1 as well as IL-2 production after activation by anti-CD3/anti-CD28 antibodies was strongly increased (Fig. 1D). More unexpectedly, despite increased phosphorylation of these key TCR downstream signaling proteins, IL-2 production was strongly reduced in the absence of ILK upon cell stimulation by SEE-loaded Raji B cells, but not by anti-CD3/anti-CD28 antibodies. These results suggest that ILK has at least 2 roles in T-cell activation: it is a negative regulator of TCR signaling and a positive regulator of the unidentified GPCR involved in SEE-mediated T-cell activation^16,17^.

There are some relevant questions that we did not address in this work. One is whether ILK affects the metabolism of T cells and in particular the activation of AKT, which is one of the ILK substrates^12^. Analysis of AKT phosphorylation status is not relevant in Jurkat T cells that are deficient for PTEN^25^ and further studies in primary T cells are needed to clarify this aspect. In addition, we did not investigate the effect of ILK inhibition on the TCR signaling cascade in primary T cells, which remains to be done in future studies.

While ILK has been partially studied in T cells, the TTK kinase, whose expression is induced by IL-2 and which is highly expressed in proliferating cells^13^, has been studied in T cells only to a very limited extent. Our study describes TTK as a protein that negatively controls the phosphorylation of several molecules downstream of TCR ligation (Fig. 3E). Like ILK depletion, TTK depletion results in a marked increase in the phosphorylation of CD3ζ, ZAP70, LAT and PLCγ1, and yet a defect in IL-2 production upon SEE activation of T cells (Figs. 3E and 4A). However, there were some differences between ILK and TTK depletion. ILK depletion significantly reduced CD3ζ levels and TCR complex expression at the plasma membrane (Fig. 1C, D), whereas TTK depletion had no effect on TCR levels (Fig. 3C). In addition, TTK appears to be needed for IL-2 production *via* both activation by anti-CD3/anti-CD28 antibodies or SEE-loaded Raji B cells, while ILK inhibits IL-2 production exclusively downstream of TCR activation by anti-CD3/anti-CD28 antibodies. Our study does not clarify why these two kinases behave differently in these two different cases of T-cell stimulation, but points out that both enzymes inhibit TCR-proximal signaling pathways, while they appear to enhance signal transduction *via* the unknown GPCR that is engaged by the superantigen in parallel with the TCR^16,17^.

The targets of these kinases in T cells are not known, but phosphoproteomic studies of TTK- and ILK-deficient T cells may reveal their identity in the future. Similarly, which factors in the TCR signaling pathway lead to the activation of these two enzymes that negatively regulate early TCR signaling? This remains a completely open question that our study does not address. In any case, these factors are likely to be among the molecules that play a negative feedback role on the amplitude of TCR-mediated signaling, e.g. tyrosine and serine phosphatases^26,27^. This hypothesis merits to be investigated in the future, especially since both ILK and TTK activation involve multiple phosphorylation events^12,28^, which can be counterbalanced by phosphatase activities.

The fact that these two enzymes are extensively studied in the context of oncogenesis, tumor survival and metastasis, and that ILK and TTK inhibitors are being developed for this purpose^29–31^, adds another dimension to our results that show that inhibition of these enzymes affects T-cell activation that may lead to their reduced anti-tumor activity. In conclusion, monitoring the activities of these inhibitors not only on tumor cell growth but also on T-cell activity will be indispensable when TTK and ILK inhibitors are proposed for chemotherapy.

## Materials and methods

### Cells

Jurkat T cells were provided by Claire Hivroz (Curie Institut, Paris) and validated by the SSTR method, and presented 88% of homology with the DSMZ ACC-282 (Leibniz). Jurkat T cells and Raji B cells (ATCC-CCL-86) were cultured in RPMI medium supplemented with 10% FCS, 2 mM L-glutamine, 50 µM β-mercaptoethanol, 100 U/ml penicillin and 100 µg/ml streptomycin. HEK293FT cells (Invitrogen, R70007) were cultured in DMEM medium supplemented with 10% FCS, 2 mM L-glutamine, 100 U/ml penicillin and 100 µg/ml streptomycin. Human primary T cells were isolated from fresh PBMC using the Pan T cell Isolation kit (Miltenyi 130-096-535), activated for 48 h with Transact (diluted 1/150) and cultured in TexMACS medium supplemented with 3% FCS, 2 mM L-glutamine, 100 U/ml penicillin and 100 µg/ml streptomycin. Human IL-2 (0.04 U/µL, Peprotech) was added to the cells on days 3 and 6, and cells were diluted every 2–3 days to 1 × 10^6^ cells/ml. Cells were used on days 10 to 12.

### Lentiviral ShRNA

ShRNA specific for CD3ζ (TRCN 0000057229), ILK (TRCN 0000279854), TTK (TRCN 0000006358) and a control, non-targeting shRNA (shNT) were purchased from Sigma-Aldrich.

### FACS analysis of human primary T cells

Human primary T cells were incubated with ILK inhibitor for 1 h and co-cultured with SEE-pulsed Raji B cells for 24 h. Dead cells were excluded with GhostDye-BV510. The antibodies used in 2 independent panels for T cell analysis were for panel 1: anti-CD3-PercP Cy5.5, CD4-BUV395, CD8-APC-Cy7, CD28-PE, CD69-APC, CD25-PE-Cy7, Ki-67-AF488. In the second panel, the PE and APC channels were used for IFNγ-PE and TNFα-APC. Data were acquired on a BD LSR Fortessa X20 using Diva software and analyzed using FlowJo software.

### Lentivirus production

The lentiviral particles were produced according to the protocol published by Tiscornia et al.^32^. Briefly, pLK0.1 plasmids encoding the shRNA, were co-transfected with the packaging plasmids pCMVDelta8.2 and the envelope plasmid pMD2G into HEK-293-FT cells via calcium chloride transfection. Six hours post transfection, the buffer was exchanged for complete DMEM and virus-containing supernatant was collected 24 and 48 h post transfection. The viral supernatants were concentrated by ultracentrifugation. Jurkat T cells were seeded in 24-well round-bottom plates at 1 × 10^6^ cells per well, and transduced in the presence of 10 µg/ml polybrene. Following 90-min centrifugation at 37 °C and 950 *g*, the lentiviral mix was replaced with complete RPMI medium. The day after, puromycin was added to the cells at 5 µg/ml, and selected cells were used 4–5 days post transduction.

### TCR signaling assays

Jurkat T cells were resuspended at 10^6^ cells/ml in complete medium and 10^6^ cells were incubated with 125 ng/ml mouse anti-CD3ε (OKT3, Biolegend) and 250 ng/ml mouse anti-CD28 (CD28.2, Biolegend) for the indicated time points (0, 5, 15 and 60 min) at 37 °C, in a water bath. At the end of each incubation, cells were immediately transferred to ice, cold PBS was added to stop the activation, the cell suspension was centrifuged at 4 °C and the cell pellet was lysed in Tris 50 mM, NaCl 150 mM, CHAPS 1% buffer supplemented with protease (Roche Complete # 04693116001) and phosphatase inhibitors (Cocktails 2 et 3, Sigma Aldrich # P5726 and # P0044).

### Western blot

The cell pellets were first lysed in Tris 50 mM pH 7.5, NaCl 150 mM, CHAPS 1% buffer supplemented with protease and phosphatase inhibitors, the lysate was clarified by centrifugation at 10,000 g for 10 min at 4 °C and the cleared supernatant was mixed 2:1 volume ratio with Laemli buffer 3x. The samples in Laemli buffer were then boiled at 95 °C for 10 min, centrifuged at 16,000 g for 5 min and loaded on Criterion 4–12% SDS-PAGE acrylamide gels (Biorad). After SDS-PAGE electrophoresis, the proteins were transferred to PVDF membranes (Biorad) using the trans-blot turbo system (Biorad). The PVDF membranes were blocked in TBS-Tween buffer (Tris 50 mM pH 7.5, NaCl 150 mM, 0.1% Tween), with 5% BSA for the immunoblots with anti-phosphorylated protein antibodies and with 5% nonfat milk for immunoblotting with other antibodies. Primary antibodies were incubated overnight at 4 °C and diluted 1000 times, except for the anti-beta-actin antibody, which was diluted 10,000 times. The primary antibodies used for immunoblotting were: mouse anti-CD3ζ (clone 6B.10.2, Santa Cruz Biotechnology), rabbit anti-ILK (clone 4G9, Cell Signaling Technology), mouse anti-TTK (N1, Thermo Fisher Scientific), rabbit anti-pZAP70 (clone 65E4, Cell Signaling Technology), rabbit anti-LAT (#06-807, Millipore), mouse anti-Lck (clone 3A5, Santa Cruz Biotechnology), mouse anti-CD3ζ (pY142) (clone K25-407.69, BD Pharmingen), rabbit anti-pLAT (#3584S, Cell Signaling Technology), rabbit anti-ZAP-70 (clone D1C10E, Cell Signaling Technology), rabbit anti-PLC*γ*1 (#2822, Cell Signaling Technology), rabbit anti-pPLC*γ*1 (#2821, Cell Signaling Technology), mouse anti-vinculin (clone hVIN-1, Sigma-Aldrich) and mouse anti-β-actin (clone AC-15, Sigma-Aldrich). All secondary antibodies were goat anti-species coupled with HRP (Jackson ImmunoResearch), diluted 20,000 times and incubated for 1 h at room temperature. The immunoblot membranes were washed extensively in TBS-Tween after each antibody incubation and the chemiluminescence signal was obtained by membrane incubation with Clarity™ Western ECL Substrate (BioRad) for 5 min. The chemiluminescence signal was acquired using a ChemiDoc™ Imaging System (BioRad) and the quantification was realized with the Image Lab software (BioRad).

### IL-2 ELISA

0.15 × 10^6^ Jurkat T cells/well were seeded in 96-well flat bottom plates and incubated with anti-CD3/anti-CD28 antibodies at 1 µg/ml final concentration for the indicated time points. For activation with SEE superantigen, Raji B cells were resuspended at 10^6^ cells/ml and 50 µl of this suspension were seeded in a well of a 96-well flat-bottom plate. Jurkat T cells transduced with shRNA lentiviral particles, or human primary T cells, were resuspended at 10^6^ cells/ml and 100 µl of T cell suspension were added to the Raji B cells in the 96-well flat-bottom plate, followed by the addition of either 50 µl SEE (Toxin Technology) solution at the indicated final concentrations. The supernatants of T cells activated by anti-CD3/anti-CD28 antibodies or of T cell-Raji B cell cocultures were harvested and IL-2 secretion was measured by ELISA using the Human IL-2 DuoSet ELISA kit (R&D Systems) according to the manufacturer’s instructions and the colorimetric reaction was read on a Tecan Infinite 200 microplate reader.

### Quantification of CD3ε levels at the cell surface by flow cytometry

Raji B cells were resuspended at 10^6^ cells/ml and 50 µl of this cell suspension were seeded in 96-well flat-bottom plates. Jurkat T cells transduced with shRNA encoding viral particles were resuspended at 10^6^ cells/ml and 100 µl of this T cell suspension were added to the Raji B cells. At the end, 50 µl of SEE (Toxin Technology) solution was added for the indicated final concentrations of SEE. Jurkat T cell culture in the absence of Raji B cells was used as control. After 6 h incubation at 37 °C in the presence of 5% CO_2_, the cell pellets were resuspended in PBS-5% BSA and labelled with a BV785-coupled anti-CD3 antibody (clone OKT3, Biolegend) for 20 min at 4 °C. The cells were then washed and incubated for 15 min in PBS-5% BSA buffer containing the dead cells exclusion marker 7-AAD and analyzed by Flow Cytometry using a Fortessa X20 instrument.

### T cell- B cell conjugate confocal microscopy

Raji B cells were resuspended at 2 × 10^6^ cells/ml in PBS and stained with Cell Trace Violet (CTV) Dye (5 µM Thermo Fisher Scientific) for 20 min at 37 °C. CTV labelling was stopped by the addition of complete RPMI medium, Raji B cells were resuspended at 2 × 10^6^ cells/ml and incubated with equal volumes of Jurkat T cell suspension at 2 × 10^6^ cells/ml for 30 min at 37 °C. Then, the cell co-culture was centrifuged, the cell pellets were resuspended at 6 × 10^6^ cells/ml in complete RPMI medium containing the SEE superantigen (100 ng/ml, Toxin Technology) and incubated at 37 °C for 30 min on Poly-L-Lysine (Sigma-Aldrich) coated slides or IBItreat micro-slides (IBIDI) for 30 min. The cells were fixed with 2% PFA prewarmed at 37 °C for 15 min and permeabilized with the permeabilization-staining buffer containing 0.2% saponin and 0.2% BSA in PBS. Primary antibodies used were: rabbit anti-LAT (#06-807, Millipore), rabbit anti-pSrc (clone D49G4, Cell Signaling Technology) and human anti-acetylated tubulin (Recombinant antibody, CurieCoreTech, Curie Institute, Paris). All antibodies were diluted 100 times. Secondary antibodies coupled with Alexa fluorochromes were diluted 100 times. The secondary antibodies were anti-mouse IgG coupled with Alexa488 (#A32766, Thermofisher), anti-human IgG coupled with Alexa594 (#709-585-098, Jackson ImmunoResearch) and anti-rabbit IgG coupled with Alexa647 (#A32795, Thermofisher). Microscopy images were acquired on an LSM 510 Zeiss confocal microscope and the image treatment, analysis and quantification were performed with ImageJ software, as previously published^33^. Briefly, the number of conjugates was counted using the ImageJ software based on the enrichment of CD3ζ in the contact region between Jurkat T and Raji B cells. The frequency of conjugation was calculated as the number of detected CD3ζ enrichments between Jurkat T and Raji B cells divided by the number of all Jurkat T cells in the imaged field of view. The average number of T cells in a field was 8.

### Quantitative RT-PCR

The total RNA was extracted from 5 × 10^6^ Jurkat T cells with the Nucleospin RNA Plus kit (Macherey-Nagel) according to the manufacturer’s instructions. One microgram of the total RNA was reverse transcribed into cDNA with the iScript cDNA synthesis kit (Biorad). Quantitative PCR was performed with Power SYBR green PCR MasterMix (Applied Biosystems) using 25ng of cDNA template. The fluorescence signal was read using the CFX-96™ Real-Time System PCR instrument (Biorad).

Sequences of qRT-PCR primers:

hGAPDH FW 5’ TGCACCACCAACTGCTTAGC 3’.

hGAPDH REV 5’ GGCATGGACTGTGGTCATGAG 3’.

TTK FWD 5’ TCAAGGAACCTCTGGTGTCA 3’.

TTK REV 5’ GGTTACTCTCTGGAACCTCTGGT 3’.

### Statistical analysis

Statistical analysis was performed with GraphPad Prism software using unpaired two-tailed Student’s t test.

## Electronic supplementary material

Below is the link to the electronic supplementary material.

Supplementary Material 1

Supplementary Material 2

Supplementary Material 3

Supplementary Material 4

Supplementary Material 5

## Data Availability

Full scans of the blots are available in Supplementary Figs. 4 and 5. All other data are available from Loredana.saveanu@inserm.fr and irini.evnouchidou@inserm.fr upon reasonable requests.
