# Supplementary material for: Integrin linked kinase and threonine tyrosine kinase modulate TCR signaling
Source: Sci Rep. 2025 Apr 24;15:14392. doi: 10.1038/s41598-025-99331-y (PMC12022052; doi:10.1038/s41598-025-99331-y)
Supplement: Supplementary file 1 — Supplementary Material 1 [file 41598_2025_99331_MOESM1_ESM.pdf]

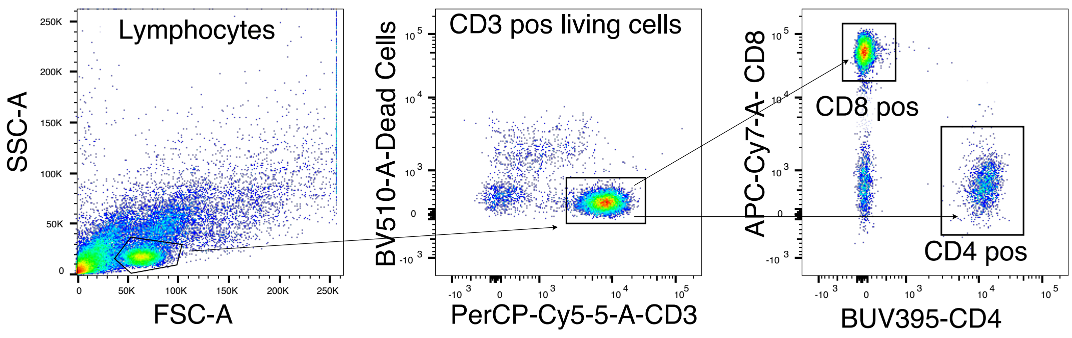

Supplementary Figure 1 Caillens et al.

Gating Strategy for Human Primary T Cell Analysis by Flow Cytometry
