# Supplementary material for: Integrin linked kinase and threonine tyrosine kinase modulate TCR signaling
Source: Sci Rep. 2025 Apr 24;15:14392. doi: 10.1038/s41598-025-99331-y (PMC12022052; doi:10.1038/s41598-025-99331-y)
Supplement: Supplementary file 2 — Supplementary Material 2 [file 41598_2025_99331_MOESM2_ESM.pdf]

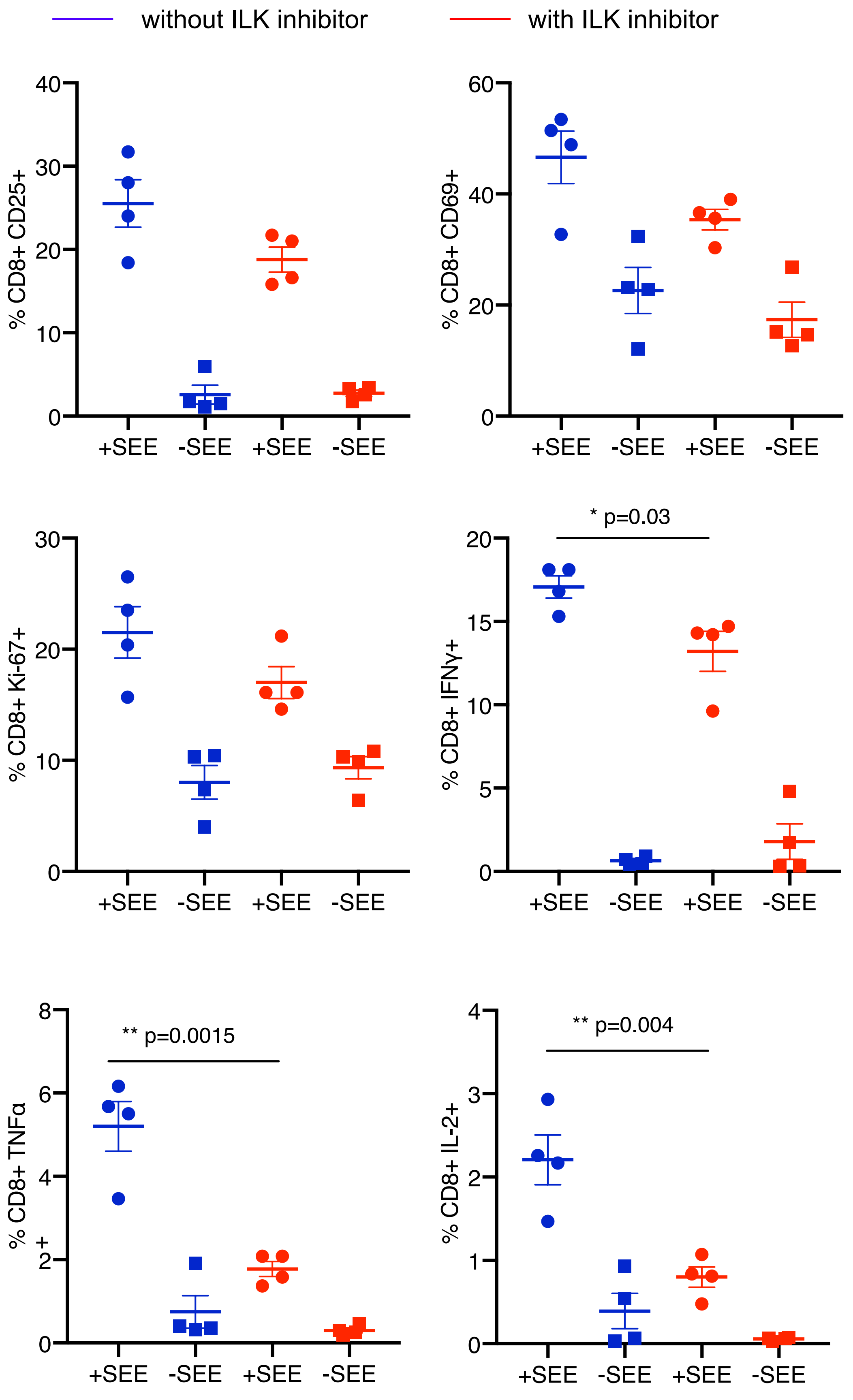

Supplementary Figure 2. Caillens et al.

Human primary T cells were preincubated with ILK inhibitor for 1 h and activated with SEE-pulsed RajiB cells in the presence of ILK inhibitor. Activation markers and cytokine production were analyzed by flow cytometry 24 hours later.
