# Supplementary material for: Integrin linked kinase and threonine tyrosine kinase modulate TCR signaling
Source: Sci Rep. 2025 Apr 24;15:14392. doi: 10.1038/s41598-025-99331-y (PMC12022052; doi:10.1038/s41598-025-99331-y)
Supplement: Supplementary file 4 — Supplementary Material 4 [file 41598_2025_99331_MOESM4_ESM.pdf]

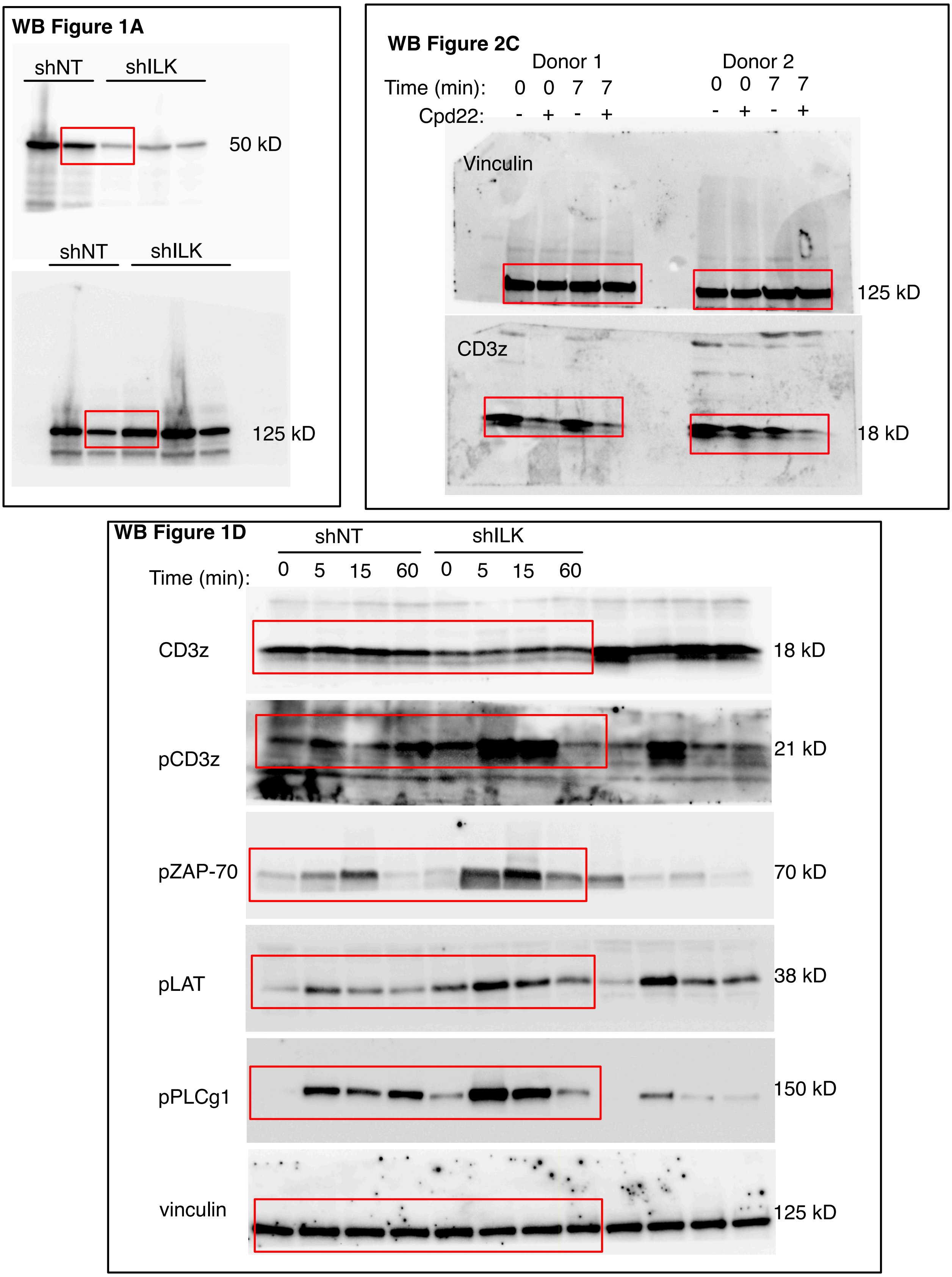

Supplementary Figure 4: Western Blots corresponding to Figure 1 and 2.
