# Supplementary figures and images for: Integrin linked kinase and threonine tyrosine kinase modulate TCR signaling
Source: Sci Rep. 2025 Apr 24;15:14392. doi: 10.1038/s41598-025-99331-y (PMC12022052; doi:10.1038/s41598-025-99331-y)

**WB Figure 3B**

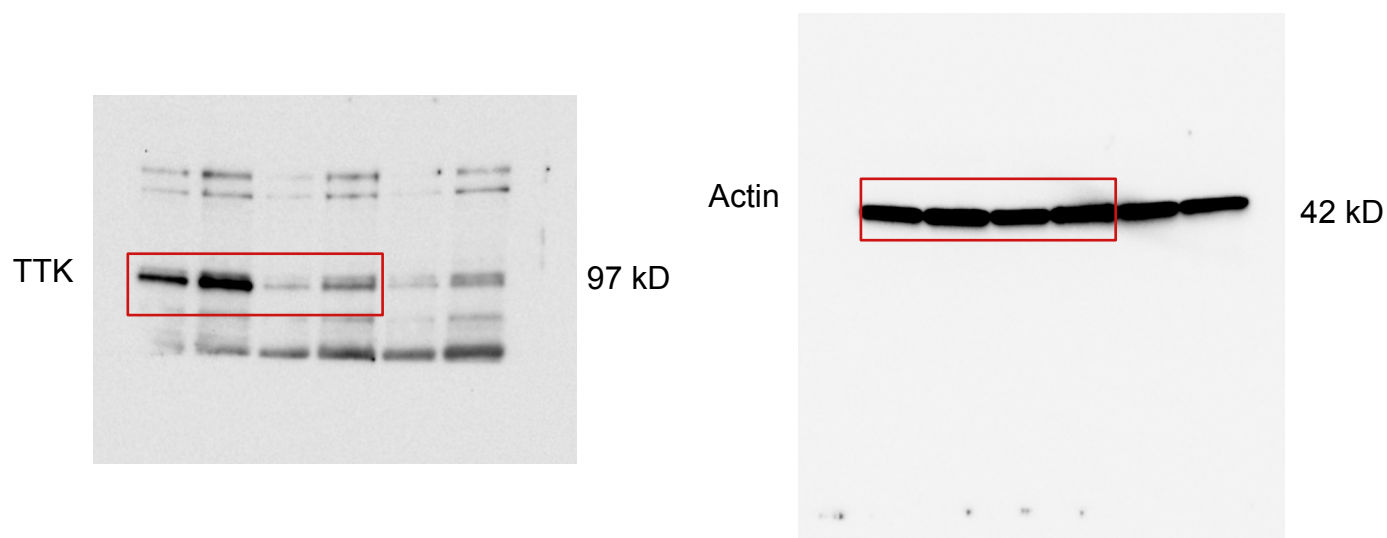

**WB Figure 3D**

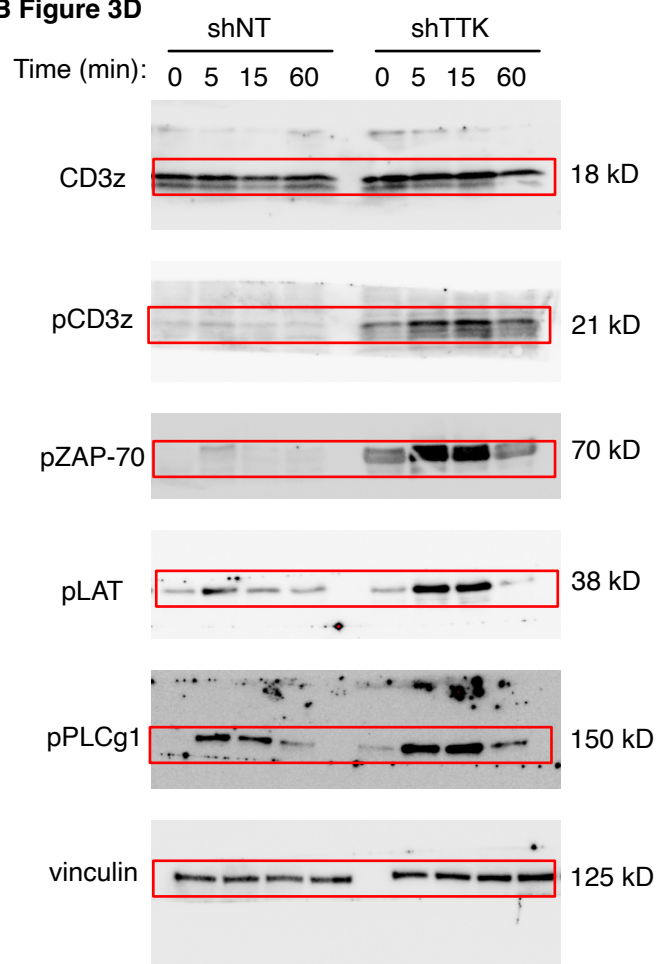

**Supplementary Figure 5: Western Blots corresponding to Figure 3.**

Supplement: Supplementary file 5 — Supplementary Material 5 [file 41598_2025_99331_MOESM5_ESM.pdf]
